# Effects of 12 Weeks of Tai Chi Chuan Training on Balance and Functional Fitness in Older Japanese Adults

**DOI:** 10.3390/sports5020032

**Published:** 2017-05-26

**Authors:** Nobuo Takeshima, Mohammod M. Islam, Yoshiji Kato, Daisuke Koizumi, Makoto Narita, Yukiko Kitabayashi, Nicole L. Rogers, Michael E. Rogers

**Affiliations:** 1 School of Health Sciences, Asahi University, Mizuho 501-0296, Japan; takeshima@alice.asahi-u.ac.jp; 2 Sport and Life Science, National Institute of Fitness and Sports in Kanoya, Kanoya 891-2393, Japan; parumon@nifs-k.ac.jp; 3 Active Aging Association, Nagoya 467-0802, Japan; yoshi-waterfalls@cam.hi-ho.ne.jp (Y.K.); dice.k.koizumi@gmail.com (D.K.); YHY02511@nifty.com (M.N.); kitabayashi.yukiko@isen.ac.jp (Y.K.); 4 Department of Public Health Science, Wichita State University, Wichita, KS 67260, USA; nicole.rogers@wichita.edu; 5 Department of Human Performance Studies, Wichita State University, Wichita, KS 67260, USA

**Keywords:** Tai Chi Chuan, effect on static and dynamic balance, functional fitness, older adults

## Abstract

The purpose of this study was to determine the effects of Tai Chi Chuan on static and dynamic balance, and functional fitness in older adults. Forty-nine volunteers were divided into an exercise group (EX: 9 men and 16 women, average age 72 ± 5 years) and control group (13 men and 11 women, average age 73 ± 6 years). The EX participated in a 12-week supervised exercise program (60 min/day, 2 days/week) that consisted of 10-min warm-up and stretching, 40-min Tai Chi Chuan exercise (long-form Yang style with 108 movements), and 10-min cool-down/relaxation exercises. The control group was asked to not change their physical activity habits. Static (sway velocity standing on firm or foam surfaces with eyes open or closed) and dynamic balance (limits of stability (LOS)), as well as functional fitness measures of body mass; upper- and lower-body strength; and flexibility, mobility, and aerobic fitness were taken before and after the intervention. After the 12-week Tai Chi Chuan exercise program, there were no improvements in any functional fitness or balance variable although components of LOS tended to increase (13.1%, *p* = 0.052). These results indicate that 12 weeks of Tai Chi Chuan exercise has no significant effect on balance and functional fitness parameters in older Japanese adults.

## 1. Introduction

The prevention of falls and the subsequent morbidity associated with fall-related injuries is important to preserve health and independence among older women. However, fall prevention strategies require a comprehensive assessment of risk factors as it is known that falls can be caused by a variety of intrinsic and extrinsic factors [1,2]. In many cases, reduced balance control is a primary risk factor for falls [1]. Reducing fall risk in older people will not only avoid increasing health care costs [3] but also, most importantly, provide older adults with a more active and enjoyable life. However, little is known about the effects of various types of balance training in the older adult population.

It has been reported that exercise reduces the risk of falls in older adults whose muscle strength has significantly deteriorated [4]. Our previous report [5] found that a customized balance exercise program is effective in improving balance and lower body strength in older adults. Improvement was also observed for limits of stability (LOS, how far one can move the center of gravity over the base of support in a given direction without stepping) in the directions that are most associated with fall-related hip fractures (i.e., left and right directions) [6]. Likewise, our other previous study of a 12-week customized balance exercise program in community-dwelling older women with poor balance ability showed similar significant improvements in LOS [7]. Therefore, it appears that such exercise programs are effective in improving balance control in older adults. 

Tai Chi Chuan has been shown to improve health status, physical fitness, mood, and emotional condition in healthy older adults as well as in stroke, multiple sclerosis, and Parkinson’s patients [8,9,10,11,12]. Rahal et al. [13] found that older expert Tai Chi Chuan practitioners had better bilateral static balance with eyes open on firm and foam surfaces compared to dancers. Likewise, Hong and colleagues [14] found that older adults who had been practicing Tai Chi Chuan for an average of 13.2 years had better balance and functional fitness compared to sedentary older adults. Tai Chi Chuan interventions have also found improvements in balance, however these have primarily been interventions lasting six months to one year [15,16,17,18], or have been performed with older adults residing in long-term care facilities [19] or in young adults [20]. Although it has been shown that those who have practiced Tai Chi Chuan for many years demonstrate better balance and that Tai Chi Chuan interventions lasting at least six months can improve balance and other parameters, it is currently unknown if Tai Chi Chuan interventions of shorter duration can improve static and dynamic balance, as well as functional fitness, in older adults who have not practiced Tai Chi Chuan in the past. The purpose of this study was to determine the effects of 12 weeks of Tai Chi Chuan training on static and dynamic balance, and functional fitness in older Japanese adults. 

## 2. Methods

Forty-nine older adults who had not previously participated in a Tai Chi Chuan program were divided into an exercise group (EX: 9 men and 16 women, average age 72 ± 5 years) and control group (13 men and 11 women, average age 73 ± 6 years) (Table 1). As a result of chi square analysis, the proportion of gender difference was not equivalent between the two groups. All subjects gave their informed consent for inclusion before they participated in the study. The study was conducted in accordance with the Declaration of Helsinki and the protocol was approved by the Ethics Committee of Nagoya City University.

### 2.1. Tai Chi Chuan Exercise Program

The EX participated in a 12-week supervised exercise program (60 min/day, 2 days/week) that consisted of 10-min warm-up and stretching, 40-min Tai Chi Chuan exercise (long-form Yang style with 108 movements), and 10-min cool-down/relaxation exercises. The control group was asked to not change their physical activity habits.

### 2.2. Measurements

Static and dynamic balance (static balance: sway velocity (SV) standing on firm or foam surfaces with eyes open or closed; dynamic balance: limits of stability (LOS) was measured before and after the intervention. LOS parameters were endpoint excursion (EPE), maximum excursion, reaction time, mean movement velocity, and directional control. Functional fitness measures—including body mass, arm curl (AC), chair stand (CS), functional reach, timed up and go, 12-min walk, back scratch, sit and reach—were taken before and after the intervention. 

### 2.3. Evaluation of Balance Ability 

A Balance Master Platform System (NeuroCom International, Clackamas, OR, USA) was used to measure static (SB) and dynamic balance (DB) [2]. SB measures are often taken while standing on different surfaces (firm or foam pad) with the eyes open or closed. In this study, the Clinical Test of Sensory Interaction for Balance using a Balance Master Platform System was used as a test of postural sway velocity (SV) that is designed to measure the influence of sensory input on balance [21]. Composite SV (SVcomp) scores were calculated based on each sway velocity condition as an index of SB. The test requires the participant to stand: (a) on a flat surface with the eyes open (SVcomp1); (b) on a flat surface with the eyes closed (SVcomp2); (c) on thick foam with the eyes open (SVcomp3); and (d) on thick foam with the eyes closed (SVcomp4). The force platform is marked to maintain consistency in foot placement. For each stance, the participant stood with their eyes at the horizon and their arms at the sides in a neutral position. Trials required 10 s of data collection. A trial was considered unsuccessful if the participant took a step or was unable to balance for the required time period without aid from a spotter. 

DB was determined using the limits of stability (LOS) assessment in which eight targets appeared around a center square at 0, 45, 90, 135, 180, 225, 270, and 315 degrees. Center of pressure (COP) appeared as a human-shaped cursor and moved as subjects shifted their weight toward an identified target, holding the position for 5 s. Each LOS trial measured endpoint (EPE) and maximum excursion (MXE). EPE ends when the COP movement first ceases progression toward the target and is expressed as a percentage of the distance to the target. Hence, a participant whose initial movement ends precisely at the target has an EPE of 100%. When initial attempts are substantially short of the target, most people initiate additional movements after the EPE is achieved. To represent this additional COP movement, an additional measurement, the MXE is used. The MXE is the maximum distance the COP is displaced toward the target over the entire duration of the trial [2]. MXE is also expressed as a percentage of the distance to the target. Composite EPE and MXE scores were calculated based on movements toward all 8 targets. The composite of reaction time (RT), movement velocity (MV), and directional control (DC) were also used.

### 2.4. Functional Fitness Test 

A battery of field tests, specifically developed for older adults, was used to assess the components of functional fitness. Using a standardized protocol, each test was conducted individually with the exception of the 12-minute walk test. In this case, participants were instructed to set their own pace and to not walk with others. These tests have been shown to have content and construct validity as well as good test-retest reliability [2,22,23,24]. 

Upper-body strength was assessed using the 30-s arm curl test (AC) [23]. On a signal, participants were instructed to flex and extend the elbow of the dominant hand, lifting a weight (men: eight-pound dumbbell, women: five-pound dumbbell) through the complete range of motion, as many times as possible in 30 s. After demonstration by the tester, a practice trial of two repetitions was given, followed by one 30-s test trial. The score was the number of repetitions completed within 30 s.

Lower-body strength was assessed using the 30-s chair stand test (CS) [23]. On a signal, participants rose to a full standing position from a chair and then returned to a seated position, and continued to complete as many full stands as possible in 30 s. After demonstration by the tester, a practice trial of one to three repetitions was given, followed by one 30-s test trial. The score was the total number of stands executed correctly within 30 s.

Balance and agility were assessed using the eight-foot up and go test (UPGO) [23] and functional reach test (FR) [22]. To perform UPGO, participants were fully seated in a chair, hands on thighs and feet flat on the floor. On a signal, participants stood from the chair, walked as quickly as possible around a cone which was placed eight feet (2.44 m) ahead of the chair, and returned to a fully seated position on the chair. Participants were told that this was a timed test and that the objective was to walk as quickly as possible (without running) around the cone and back to the chair. After demonstration by the tester, participants were given one practice trial and two test trials. Performance time was recorded in units of 0.1 s and the best score was used for analysis. To perform FR, a scale (graduated in cm) was hung from a wall at a height suitable for the participant. After demonstration by the tester, participants were given one practice trial and two test trials. The participant stood by the wall with feet together, hands clasped and both arms raised in front horizontally and held at the 0 cm level of the scale. On a signal, the participant leaned forward, moving the hands forward along the scale as far possible while keeping the heels in contact with the ground. Performance was assessed as the maximal distance the participant could reach forward beyond arms’ length and without taking a step. The best score of the two test trials was used to evaluate performance.

Upper-body flexibility was assessed using the back scratch test (BS) [23]. Participants placed the preferred hand behind the same side shoulder with the forearm pronated and fingers extended. They placed the other hand behind the back, forearm supinated, reaching up in an attempt to touch or overlap the extended middle fingers of both hands. After demonstration by the tester, participants were asked to determine the preferred hand, and were given two practice trials, followed by two test trials. The score was the number of cm the middle fingers were short of touching (minus score) or overlapped each other (plus score). The best score of test trials was used to evaluate performance.

Lower-body flexibility was assessed using the chair sit and reach test (SR) [23]. Participants sat on the edge of a chair with one leg bent and the other leg extended straight in front with the heel on the floor. Without bending the knee, participants slowly reached forward, sliding the hands down the extended leg in an attempt to touch the toes. After demonstration by the tester, participants were asked to determine the preferred leg and were given two practice trials on that leg, followed by two test trials. The score was the number of cm short of reaching the toes (minus score) or reached beyond the toes (plus score). The best score of two test trials was used to evaluate performance.

Cardio-respiratory fitness was assessed by performing the 12-min Walk Test (WT) that assessed the maximum distance walked in 12 min around a 60-m rectangular course marked into 5-meter segments [25,26]. The score was the total number of meters walked in 12 min. 

After 12 weeks, all measurements were repeated in each participant. All tests were conducted by the same tester during pre and post testing. 

### 2.5. Statistical Analysis

Data were analyzed using analysis of variance (ANOVA) with repeated measures for the main effects of group, time and interaction (group × time). At baseline, significant differences were noted in parameters between groups so the influence of the initial level of these parameters was examined using analysis of covariance (ANCOVA). Effect size (ES) was also calculated for each test [27]. Cohen’s definition of small, medium, and large ES (ES = 0.2, 0.5, and 0.8, respectively) was used Cohen [28]. The significance level was set at α = 0.05.

## 3. Results

At baseline, significant differences were noted in AC, CS, and DCL between groups so the influence of the initial level of these parameters was examined using analysis of covariance (ANCOVA) (Table 2). 

After the 12-week Tai Chi Chuan exercise program, there were no improvements in any variable although EPEcomp tended to increase (13.1%, *p* = 0.052) (Table 3).

## 4. Discussion

This study evaluated the effects of Tai Chi Chuan on static and dynamic balance, and functional fitness in older Japanese adults. Tai Chi Chuan is a Chinese traditional mind-body exercise with a low to moderate exercise intensity [29]. From a systematic review and meta-analysis [11], Tai Chi Chuan is helpful for healthy adults in improving various aspects of cardiorespiratory fitness, specifically stroke volume, forced vital capacity, maximum minute ventilation, and peak VO_2_. However, the present study did not show any benefits of Tai Chi Chuan exercise on any parameters of functional fitness or static and dynamic balance. The lack of improvements is consistent with other meta-analyses that concluded there is a paucity of data to support Tai Chi Chuan as an effective intervention for improving balance and functional status, or reducing fall risk and the fear of falling [30,31]. One of the reasons for the lack of improvements may be that Tai Chi Chuan is difficult to perform for older Japanese adults who are not accustomed to this activity. Although an advantage of Tai Chi Chuan is that it can be performed without any special exercise equipment, it may be an activity that is very difficult to learn over a period of 12 weeks. Based on the observations of the researchers, it appeared to take participants 4–8 weeks to become familiar with the movements. Therefore, an intervention of much longer length may be necessary to show any improvements.

It has previously been reported that Tai Chi Chuan improves dynamic balance (LOS) but that was after a program lasting 48 weeks [17]. Another report [16] found that a six-month Tai Chi Chuan training program improved measures of functional balance. While the current 12-week program did not result in any changes, our previous studies utilizing Standing Strong, a customized balance training program [32], found significant improvements in functional fitness as well as static and dynamic balance after only 12 weeks in older adults with poor balance [7] as well as in healthy older adults [5]. Therefore, a customized balance program that directly targets the physiological systems involved in balance appears to be more beneficial in the short-term. Future studies of these interventions being conducted in similar groups over a period of 6–12 months with monthly assessments of functional fitness and balance should elucidate the differences in dose–response relationships for these programs.

Tai Chi Chuan is considered to be one of the martial arts. As such, a basic premise of Tai Chi Chuan is that if one is attacked by a partner or enemy then the position must be held without moving the position of the center of gravity. Furthermore, Tai Chi Chuan training is performed on a solid surface. In our customized balance exercise program for older adults, participants move the center of gravity on an unstable surface (i.e., foam pads). As a result, participants are challenged to control their center of gravity as it moves towards the limits of stability. In contrast, Tai Chi Chuan does not provoke such levels of instability. Therefore, given that the martial arts focus on maintaining a solid, upright position with little challenge to the center of gravity, it may be difficult to improve postural balance. Again, further study is needed to compare the long-term outcomes of these exercise programs. 

Limitations of this study should be considered. Outcomes of this study were restricted to balance and functional fitness and did not consider other elements that may be affected by Tai Chi Chuan such as psychological parameters. For example, it is possible that balance confidence or fear of falling, quality of life, and elements of spirituality were enhanced but these were not measured. In addition, there are limitations in generalizability. This study showed no significant improvements associated with the 12-week Tai Chi Chuan intervention but the results were demonstrated in only older Japanese adults and may not be applicable to other populations such as those in Western societies. 

## 5. Conclusions

In conclusion, while Tai Chi Chuan interventions lasting six months to one year have resulted in improvements in balance and functional fitness, it does not appear that 12 weeks of Tai Chi Chuan training is sufficient to improve these parameters in older Japanese adults. This may be due to the difficulty in learning proper Tai Chi Chuan techniques and/or that the physiologic systems that control balance are not adequately challenged. In order to improve balance and functional fitness over a short period of time (i.e., 12 weeks), it is recommended that older adults participate in other training programs that have been shown to improve balance and functional fitness.

## Figures and Tables

**Table 1:** Physical characteristics of the participants.

	Tai Chi Group (*n* = 25)	Control Group (*n* = 24)	Group Difference
Age (years)	72.2 ± 5.0	73.1 ± 6.7	N. S.
Height (cm)	156.1 ± 8.2	158.1 ± 6.0	N. S.
Body mass (kg)	57.1 ± 11.3	57.1 ± 7.6	N. S.
BMI (kg/m^2^)	23.4 ± 4.1	22.8 ± 2.2	N. S.

Note: BMI: body mass index (body mass/height^2^); N.S.: not significant between groups.

**Table 2:** Effect of Tai Chi Chuan on functional fitness in older adults.

		Pre	Post	Change (%)	Effect Size	Group Effect	Time Effect	Group × Time
AC ^a^ (reps)	Tai Chi group	32.2 ± 3.7 *	32.5 ± 3.6	1.1	0.08	N.S.	N.S.	N.S.
	Control group	26.8 ± 4.3 *	27.3 ± 3.9	1.9	0.12			
CS ^a^ (reps)	Tai Chi group	32.4 ± 4.6 *	32.5 ± 4.7	3.5	0.02	N.S.	N.S.	N.S.
	Control group	26.7 ± 5.5 *	27.2 ± 5.5	1.9	0.09			
UPGO ^b^ (s)	Tai Chi group	4.4 ± 0.6	4.3 ± 0.5	3.4	0.17	N.S.	F = 14.554 (*p* < 0.05)	N.S.
	Control group	4.9 ± 1.3	4.7 ± 1.4	2.7	0.15			
SR ^b^ (cm)	Tai Chi group	14.7 ± 7.7	14.9 ± 7.8	0.9	0.03	N.S.	F = 5.026 (*p* = 0.030)	N.S.
	Control group	8.1 ± 15.4	8.3 ± 15.1	2.8	0.01			
BS ^b^ (cm)	Tai Chi group	−5.4 ± 12.2	−5.1 ± 12.1 12.1 ± 12.1	4.8	0.03	N.S.	N.S.	N.S.
	Control group	−7.7 ± 13.0	−7.3 ± 12.8	0	0.03			
FR ^b^ (cm)	Tai Chi group	36.2 ± 4.2	36.4 ± 4.1	0.5	0.05	N.S.	F = 9.312 (*p* < 0.05)	N.S.
	Control group	35.6 ± 4.4	36.4 ± 5.9	2.2	0.14			
WT ^b^ (m)	Tai Chi group	1123 ± 107	1184 ±103	5.4	0.57	N.S.	F = 28.077 (*p* < 0.05)	N.S.
	Control	1160 ± 142	1224 ± 142	5.5	0.45			

^a^ *p* value from repeated-measures analysis of covariance (ANCOVA); ^b^ repeated-measures analysis of variance (ANOVA); * Significantly different from the baseline value in the same group; *p* < 0.05; N.S.: not significant; AC: arm curl test; CS: chair stand test; UPGO: up and go test; SR: sit and reach test; BS: back scratch test; FR: functional reach test; WT: 12-min walk test.

**Table 3:** Effect of Tai Chi Chuan on static and dynamic balance in older adults

	Parameter	Group	Pre	Post	Change (%)	Effect Size	Group Effect	Time Effect	Group × Time
Dynamic balance	RTcomp ^b^ (s)	Tai Chi	0.86 ± 0.21	0.81 ± 0.19	5.8	0.24	N.S.	N.S.	N.S.
		Control	0.84 ± 0.32	0.85 ± 0.25	1.2	0.03			
	MVLcomp ^b^ (deg/s)	Tai Chi	4.30 ± 1.99	4.11 ± 1.23	4.4	0.10	N.S.	N.S.	N.S.
		Control	4.58 ± 2.24	4.04 ± 1.44	11.8	0.24			
	EPEcomp ^b^ (%)	Tai Chi	64.6 ± 13.6	73.1 ± 9.4	13.1	0.63	N.S.	F = 15.614 (*p* < 0.05)	N.S.
		Control	60.1 ± 13.2	65.5 ± 9.7	9.0	0.41			
	MXEcomp ^b^ (%)	Tai Chi	79.3 ± 10.6	89.0 ± 7.2	12.3	0.92	F = 5.676 (*p* < 0.05)	F = 43.500 (*p* < 0.05)	N.S.
		Control	73.3 ± 13.0	82.3 ± 9.4	12.3	0.69			
	DCLcomp ^a^ (%)	Tai Chi	73.0 ± 8.8 *	74.8 ± 5.0	2.58	0.21	N.S.	F = 64.474 (*p* < 0.05)	N.S.
		Control	66.1 ± 8.6 *	73.9 ± 7.6	11.9	0.91			
Static balance	SVcomp ^b^ (deg/s)	Tai Chi	1.16 ± 0.38	1.09 ± 0.34	6.0	0.19	N.S.	N.S.	N.S.
		Control	1.14 ± 0.35	1.12 ± 0.38	1.8	0.06			

^a^ *p* value from repeated-measures analysis of covariance (ANCOVA); ^b^ repeated-measures analysis of variance (ANOVA); * Significantly different from baseline value in the same group, *p* < 0.05, N.S., not significant; RTcomp: reaction time composite score; MVLcomp: movement velocity composite score; EPEcomp: endpoint excursion composite score; MXEcomp: maximum excursion composite score; DCLcomp: directional control composite score; SVcomp: sway velocity composite score; composite scores were calculated based on movements toward eight targets.
